# Severe fatigue is associated with diminished lung function and elevated Galectin-9 levels in early systemic sclerosis

**DOI:** 10.3389/fimmu.2025.1655414

**Published:** 2025-09-05

**Authors:** Charmaine van Eeden, Maryam Rezaeifar, Muhammad Elezzabi, Desiree Redmond, Robert Gniadecki, Andrew Abey, Erin Reynolds, Dalton Sholter, Shima Shahbaz, Shokrollah Elahi, Lamia Khan, Andrew Mason, Marvin J. Fritzler, Douwe J. Mulder, Murray Baron, Maggie J. Larché, Janet Pope, May Choi, Sabrina Hoa, Carter Thorne, Elena Nethchiporouk, Jan Willem Cohen Tervaert, Mohammed S. Osman

**Affiliations:** ^1^ Department of Medicine, University of Alberta, Edmonton, AB, Canada; ^2^ School of Dentistry, University of Alberta, Edmonton, AB, Canada; ^3^ Cumming School of Medicine, University of Calgary, Calgary, AB, Canada; ^4^ Department of Internal Medicine, University of Groningen, Groningen, Netherlands; ^5^ Department of Medicine, McGill University, Montreal, QC, Canada; ^6^ Alberta Health Services – Calgary Zone and Cumming School of Medicine, University of Calgary, Calgary, AB, Canada; ^7^ Division of Rheumatology, University of Western Ontario, London, ON, Canada; ^8^ Department of Medicine, University of Montreal, Montreal, QC, Canada; ^9^ Division of Rheumatology, University of Toronto, Toronto, ON, Canada

**Keywords:** systemic sclerosis, fatigue, galectin-9, pulmonary function, vascular remodeling

## Abstract

**Introduction:**

Symptoms resembling myalgic encephalomyelitis/chronic fatigue syndrome (ME/CFS) frequently affect patients with rheumatic diseases, but little is known about their frequency and disease manifestations, particularly in systemic sclerosis (SSc) patients. We sought to determine if severe fatigue in SSc patients with early disease (< 7 years) is associated with increased disability, inflammation and fibrosis.

**Methods:**

In this exploratory cross-sectional study, 51 SSc patients were recruited locally (UofA cohort). Disability, disease damage accrual, inflammatory markers and, indicators of fibrotic and vascular complications (e.g. lung function, nailfold capillaroscopy) were compared between patients with and without severe fatigue. Fatigue was assessed using validated questionnaires (e.g. FACIT, MFI) and ME/CFS criteria. Findings were further corroborated in the national CSRG (Canadian Scleroderma Research Group) SSc cohort (n=126).

**Results:**

SSc patients with severe fatigue had significantly increased disability, reduced lung function capacity, and elevated Galectin-9 levels when compared to patients without fatigue. Galectin-9 levels correlated with reduced pulmonary function, and increased disease damage accrual. Further analysis in the UofA cohort suggested that indictors associated with disease progression such as reduced nailfold capillary density, and elevated VEGF, LTα and IL-16 were present in severely fatigued patients.

**Discussion:**

Severe fatigue in SSc patients is associated with increased disability, reduced pulmonary function and increased vascular remodeling. We propose that ME/CFS-like symptoms in patients with SSc may be indicative of sub-clinical inflammation and fibrosis. Further studies are required to determine whether Gal-9,may be a useful tool for the stratification of SSc patients - particularly those with severe fatigue resembling ME/CFS.

## 1 Introduction

Systemic sclerosis (SSc) is a life-threatening disease of unclear etiology characterized by immune dysregulation, vasculopathy, and fibrosis of the skin and internal organs (1). Among the various contributors to disability in SSc, we have come to learn that non-specific symptoms such as fatigue have a profound effect on quality of life and loss of income (2). Fatigue in SSc is common, with rates ranging between 48-79% (2); however, it is still largely ignored in the clinic as its mechanisms are poorly understood, and treatment options are lacking.

The knowledge gap related to fatigue stems from inconsistent use of objective patient reported outcomes which only span a short period of time (1 week, e.g. multifunctional fatigue inventory (MFI), functional assessment of chronic illness therapy (FACIT), short form 36 (SF36)). In many patients with SSc, severe fatigue is long lasting and is associated with other symptoms (e.g. widespread pain, post-exertional malaise and cognitive dysfunction), which often antecede a diagnosis of SSc (2). Hence, a more consistent definition of severe fatigue that is more applicable to SSc is needed, particularly in translational research studies. To address this, we applied the international consensus criteria for myalgic encephalomyelitis/chronic fatigue syndrome (ME/CFS) to patients with SSc (3). In these criteria, severe fatigue must be present for at least six (6) months. In addition, non-refreshing sleep and post exertional malaise is present accompanied with other symptoms such as brain fog (or cognitive dysfunction), and diffuse body pain resembling fibromyalgia. Although the ME/CFS criteria have not been validated in SSc, many patients (40-60%) with various inflammatory diseases (e.g. ANCA-associated vasculitis, SSc and post-acute COVID syndrome (PACS)) a large proportion of patients (40-60%) met them (4–7). Further to this, our approach in these studies allowed us to uncover numerous biological differences between fatigued and non-fatigued patients with underlying chronic inflammatory diseases (4–7).

Previous studies have suggested that fatigue in SSc may be associated with interstitial lung disease (ILD) – although little granularity related to the potential associated mechanisms have been proposed (2, 8). SSc-ILD is present in a large proportion of both diffuse cutaneous SSc (dcSSc) and limited cutaneous (lcSSc) patients and is associated with significantly lower survival rates at 10 years (9). In patients with SSc-ILD, several biomarkers (e.g. anti-topoisomerase 1, C-reactive protein (CRP), Interferon gamma induced protein 10 (IP-10, CXCL10) Interleuken-23 (IL-23), and Interleukin-6 (IL-6)) have been suggested to be linked with disease progression in both dcSSc and lcSSc (9–12). Of these markers, CRP, CXCL10 and IL-6, have also been shown to be associated with ME/CFS in other inflammatory conditions such as post-acute covid syndrome (PACS) (5). Cytokines are however, ubiquitous in nature, with variations in expression being affected by factors such as diet, environment, time of day, and other factors related to sample processing (13, 14). Hence, more stable biomarkers that are linked to associated mechanisms are optimal. Galectin-9 (Gal-9), an integral immunomodulatory protein expressed in most cell types, may be more suitable (15). Gal-9 has been linked to increased organ involvement and mortality in both lcSSc and dcSSc patients (16, 17), rapidly progressive ILD in dermatomyositis patients (18), and has been proposed as a biomarker in patients with PACS complicated by ME/CFS (5).

In this study, we aimed to determine if severe fatigue resembling ME/CFS is associated with ILD in SSc patients, with the ultimate goal of linking our clinical observations with previously identified markers of diminished lung function in SSc patients. Together, our approach may provide a platform for future studies which could provide additional mechanistic insights related to how fatigue may be linked with fibrotic complications of SSc. To achieve this, we utilized two independent SSc cohorts, including both lcSSc and dcSSc patients with a < 7-year disease duration from the onset of non-Raynaud symptoms.

## 2 Materials and methods

### 2.1 University of Alberta cohort

SSc patients over the age of 18 years referred to the SSc Clinic at the University of Alberta between June 2019 and April 2024, were asked to participate. All patients met the 2013 European Alliance of Associations for Rheumatology/American College of Rheumatology (ACR/EULAR) classification criteria (19), for SSc. Patients with a hemoglobin <100 g/L, or iron levels and/or thyroid hormone levels outside the normal reference ranges and those requiring supplemental oxygen were excluded. Patients were sequentially recruited for this study. We selected only patients with a disease duration of less than 7 years. The study was approved by the University of Alberta Research Ethics Board (Pro00085583, Pro00090050), and all research was performed in accordance with the recommended guidelines. Written informed consent was obtained from all the study participants. Patients were excluded if they were treated with an autologous stem cell transplant, had a history of cancer, SLE/SSc overlap, autoimmune thyroid disease or diabetes.

All patients were tested for anti-nuclear and SSc-specific autoantibodies (20) (as summarized in **Supplementary Table 1**) by Mitogen Diagnostics Corp., (Calgary, Canada). Laboratory parameters such as complete blood cell counts, C-reactive protein (CRP), vitamin B12 and thyroid-stimulating hormone (TSH), direct antiglobulin test (DAT) and anti-tissue transglutaminase antibodies (ATTG) levels were measured by Alberta Precision Laboratories (Edmonton, Canada). The presence of ILD was determined via high resolution chest tomography (HRCT), based on the presence of patterns as described in patients with SSc, including: non-specific interstitial pneumonia (NSIP), ground glass opacities (GGO) and usual interstitial pneumonia (UIP). The severity of ILD was estimated using pulmonary function test (PFT) parameters. All patients were screened for pulmonary arterial hypertension PAH) and left ventricular dysfunction using transthoracic echocardiography. Patients with suspected PAH, subsequently had a confirmatory right heart catheterization.

Patients underwent contemporaneous clinical and NVC assessments at the time of enrolment. All images were captured using a 200X Video Capillaroscope (DS Medica, Milan, Italy). Collected NVC parameters included: mean capillary density (number of capillaries per mm averaged over 8 digits), apical capillary diameter (µm), and microhemorrhages (number of hemorrhages per digit, averaged over the 8 digits), as previously described (21, 22). Plasma samples (EDTA) were collected, processed within 3 hours and stored at -80 °C until use.

### 2.2 CSRG cohort

The CSRG registry is an ongoing longitudinal study of adult (>18 years) scleroderma patients recruited by rheumatologists from 14 sites across Canada and 1 in Mexico. All sites have the approval of their institutional review boards and patients provide written consent. The study was approved by the ethics committee at the Jewish General Hospital in Montreal, Canada. Data is collected at yearly visits using a standardized data collection protocol which includes clinical (both patient and physician reported) and laboratory data and is recorded in a customized database. We included 126 unique patients, all of whom fulfilled the 2013 ACR/EULAR criteria for SSc. These patients were selected based on their disease duration (< 7 years). Patients were excluded if they had cancer, SLE/SSc overlap, autoimmune thyroid disease, diabetes, or a hemoglobin <100 g/L. ILD and lung function screening for the CSRG have been previously described (23). Serum was collected at the baseline registry visit and sent to a central laboratory at the University of Calgary, where samples were aliquoted and stored at −80°C.

### 2.3 Patient involvement

Patients were not involved in designing the research question or outcome measures of this study. Results from the study, will be made available to all participants, and will be disseminated at both scientific and patient-based meetings, to facilitate increased patient and public involvement in future studies.

### 2.4 Questionnaires

Diagnosis of ME/CFS was based on the Canadian Consensus Criteria (24) where PEM and non-refreshing sleep are a required feature and the duration of fatigue is ≥ 6 months. Enrolled patients were assessed for the presence of fatigue and disability using previously validated questionnaires (DePaul symptom questionnaire (DSQ-2), the functional assessment of chronic illness therapy (FACIT), multidimensional fatigue inventory (25), health assessment questionnaire (HAQ) disability index (DI), and the 36-Item Short Form Survey (SF-36) (**Supplementary Table 2**). Comorbidities (e.g., anxiety, sleep disturbances, and depression) were also assessed using validated questionnaires (**Supplementary Table 2**).

In the CSRG cohort data related to the FACIT-fatigue scale was collected. We assessed the relationship between FACIT scores and the presence of ME/CFS in the UofA cohort, and found good accuracy, sensitivity and specificity and an accuracy of 90%, using the FACIT fatigue cutoff of <30 for the identification of ME/CFS (**Supplementary Figure 1**).

### 2.5 Cytokine, chemokine and growth factor panels

Biomarkers were measured using the V-Plex proinflammatory panel 1, cytokine panel 1, chemokine panel 1, Th17 panel 1, Angiogenesis Panel 1, and Vascular panel injury 2 (Meso Scale Discovery) (MSD, Rockville, MD, USA), according to the manufacturer’s instructions. The plasma samples were centrifuged at 2,000 × g for 10 minutes and then appropriately diluted according to the specific guidelines provided for each assay. Samples were run in duplicate.

### 2.6 Gal-9 ELISA

The Gal-9 ELISA (DuoSet Human Galectin-9 DY2045) was performed according to manufacturer’s instructions, using the recommended Ancillary Reagent Kit (DY008B) (R&D Systems, Minneapolis, MN, USA). Due to sample availability, plasma samples were used for the UofA cohort and serum samples for the CSRG comparison cohort. In house testing showed that the use of both serum and plasma resulted in similar Gal-9 levels being detected using the aforementioned ELISA kit (**Supplementary Figure 2**). Samples were run in duplicate.

### 2.7 Statistical analysis

All statistical analyses were completed using Stata 18 (StataCorp) (26). For univariate analysis: continuous variables, median and interquartile range are indicated with the exact p-value as determined by the Wilcoxon Rank Sum Test; for categorical variables, chi-square analysis was carried out with Fisher’s exact test. Variables are indicated as the total number positive responses and the percentage of positive responses within the population. Spearman’s rank-order was used for correlation coefficient analysis, with Bonferroni correction. Individual correlations were visualized using linear prediction plots with confidence intervals and overlaid scatterplot.

This was an exploratory study, and as such no formal sample size was calculated. The sample size for both cohorts was dependent on the availability of both blood samples and fatigue data in patients whom met the inclusion criteria.

### 2.8 Role of the funding source

The funders had no involvement in the study design, or the collection, analysis and interpretation of the data.

## 3 Results

### 3.1 Characteristics of the SSc UofA cohort

This cross-sectional study included 51 SSc patients from the UofA cohort; 45 lcSSc and 6 dcSSc. Using ME/CFS classification criteria 25 patients were classified as fatigued (SSc-F) and 26 non-fatigued (SSc-NF). Comparably, FACIT (<30) classified 22 patients as fatigued and 28 as non-fatigued. As the ME/CFS classification criteria account for symptoms over a period of six months or more, compared to only a week for the FACIT. Fatigue grouping in the UofA cohort was based on ME/CFS criteria to allow a more robust and homogeneous comparison of factors associated with fatigue that may be linked with potential mechanisms.

Disease duration was similar between both groups, with the median duration being 1–2 years from first non-Raynaud manifestation (**Supplementary Table 3**). Age and sex did not substantially differ between the groups (**Supplementary Table 3**). Importantly, disease-associated damage parameters including indicators of skin fibrosis, digital ulcers, joint contractures, puffy fingers, and organ and gastrointestinal involvement were similar. (**Supplementary Tables 3**, **4**). The frequency of PAH and ILD did not differ between SSc-F and SSc-NF patients. A trend, however, was evident for increased disease damage accrual (Scleroderma Clinical Trials Consortium Damage Index (SCTC-DI)), in SSc-F patients, when compared to SSc-NF patients (p=0.050). Autoantibodies, CRP, TSH, markers of autoimmune anemia (e.g. DAT) and diabetes (hemoglobin A1c) and vitamin B12 levels were also similar between the two groups (**Supplementary Tables 3**, **4**). In addition, use of immunomodulatory and vasodilator medication classes did not differ between groups. (**Supplementary Table 3**).

### 3.2 Characteristics of the SSc CSRG cohort

126 patients in the CSRG cohort met our inclusion criteria: 81 patients with lcSSc and 45 with dcSSc (**Supplementary Table 5**). Median disease duration was 2.8 years for lcSSc patients and 1.5 years for dcSSc patients. Among them, 45/126 (35.7%) patients were classified as having ME/CFS based on a FACIT fatigue score of <30 (CSRG-F), whereas, 81/126 (64.2%) were non fatigued (CSRG-NF). There were significantly more females in the CSRG-F group (93.3%) than in the CSRG-NF group (76.4%) (p=0.017) (**Supplementary Table 5**). There were no differences between CSRG-F and CSRG-NF patients with regards to age, BMI, skin involvement (modified Rodnan skin score (mRSS)), ILD, digital ulcers, medication use, disease duration or SSc disease type (**Supplementary Table 5**). CRP levels were however higher in CSRG-F patients (p=0.043).

### 3.3 Severe fatigue is strongly associated with disability in systemic sclerosis

**Figure 1:**
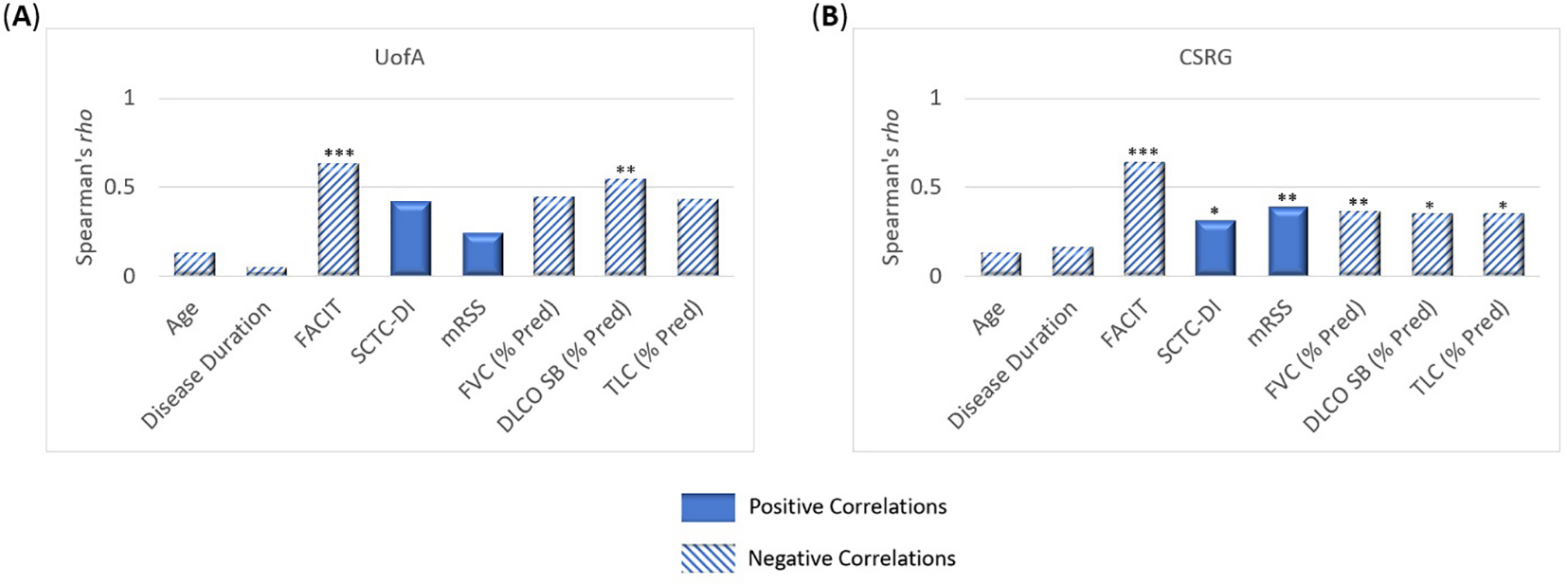
Correlates of disability (HAQ-DI) in systemic sclerosis patients. Bonferroni corrected Spearman correlations showing the relationship between disability and disease associated parameters in both the **(A)** UofA (n=49) and **(B)** CSRG (n=126) cohorts. In both the UofA and CSRG cohorts, fatigue (FACIT) was the strongest correlate of disability. DLCO SB (% Pred) also correlated with disability in both cohorts. In the CSRG cohort, disability also correlated with disease accrual (SCTC-DI), skin involvement (mRSS), TLC (% Pred), and FVC (% Pred). * p <0.05, ** p <0.01, *** p <0.001.

In the UofA cohort, the incidence of cognitive failure (CFQ) (p=0.004), anxiety (HADS) (p=0.047) and depression (HADS) (p=0.010) were higher in SSc-F patients (**Supplementary Table 3**). Both the SF-36 physical component score (PCS) and the mental component score (MCS) were significantly lower in the SSc-F group (p<0.001) (**Supplementary Table 3**). Disability scores (HAQ-DI) were significantly higher in SSc-F patients (0 vs 0.75; p<0.001) (**Supplementary Table 3**). In addition, there was a significant difference in the employment status of SSc-F patients with 32.0% being on disability welfare, compared to none in the SSc-NF group (p=0.010) (**Supplementary Table 4**). Correlate analysis showed that fatigue (FACIT) was the strongest correlate of disability (HAQ-DI) (rho:0.634, p<0.001) in the UofA cohort, even when controlling for SSc disease-specific indices (**Figure 1A**, **Supplementary Figure 3A**). DLCO SB (% Predicted) was also correlated with disability (rho:0.542, p=0.008).

Patient reported co-morbidities were assessed in the CSRG cohort. Quality of life (SF-36) was significantly reduced in CSRG-F patients (p<0.001), and disability (HAQ-DI) was significantly higher (p<0.001) (**Supplementary Table 5**). Correlate analysis showed that fatigue (FACIT) outweighed measures of SSc disease with regards to patient disability (HAQ-DI) (rho:0.64, p<0.001) (**Figure 1B**, **Supplementary Figure 3B**). The CSRG cohort included more dcSSc patients (35.7%) than the UofA cohort (11.7%), and thus unsurprisingly significant correlations with disability, were also observed for skin involvement (p=0.002), damage accrual (p=0.049), as well as lung function (p<0.05), though rho values were moderate (<0.4) (**Figure 1B**, **Supplementary Figure 3B**).

### 3.4 Systemic sclerosis patients with severe fatigue have diminished lung function indices

**Figure 2:**
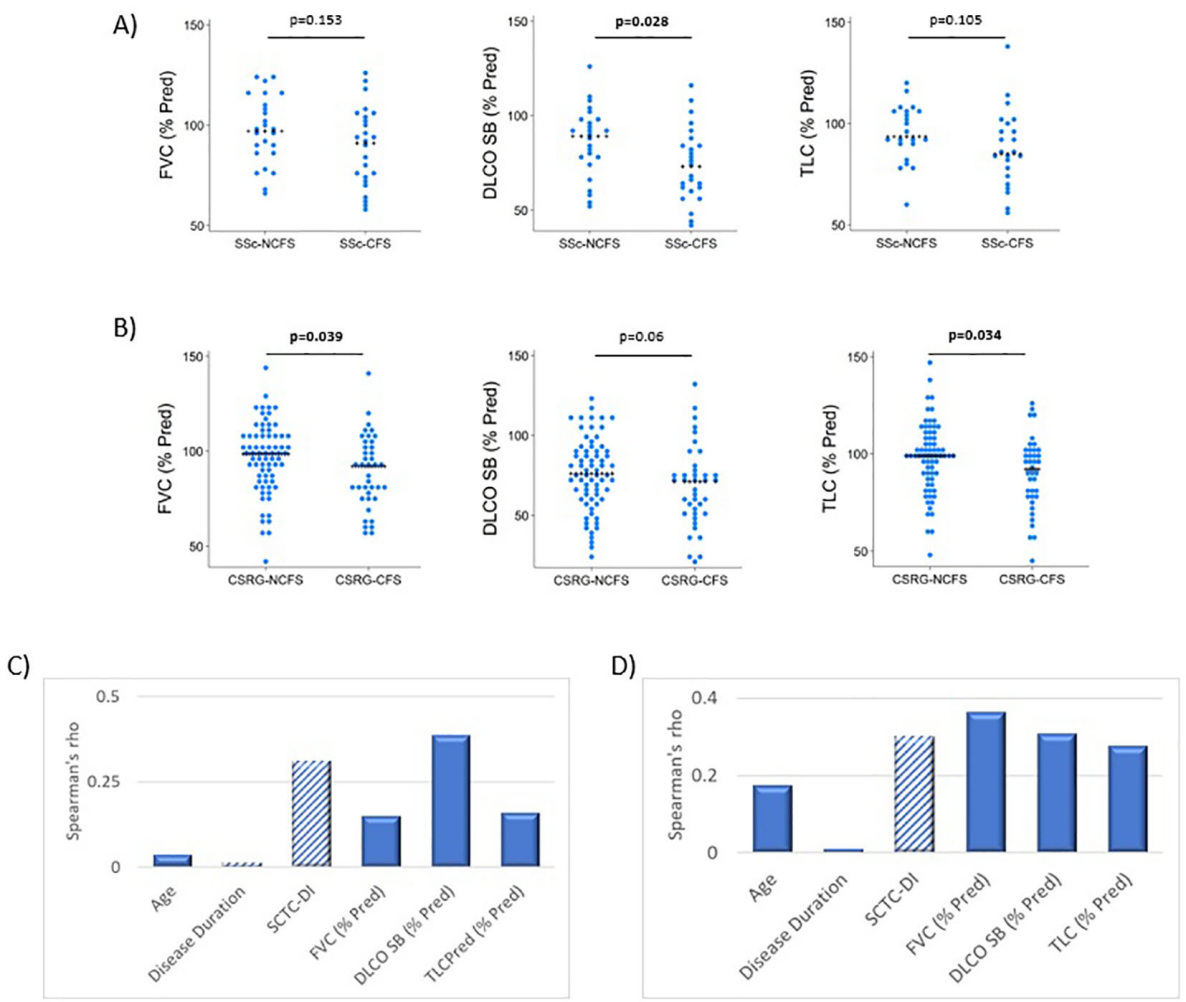
Lung function parameters associated with severe fatigue systemic sclerosis patients. **(A)** In the UofA cohort, DLCO SB (p=0.028) was significantly reduced in SSc-F (n=25) compared to SSc-NF (n=25) patients. P-values determined by Wilcoxon rank-sum test. **(B)** Severe fatigue is associated with diminished lung function in the CSRG comparison cohort, both FVC (p=0.039) and TLC (p=0.034) were significantly lower in CSRG-F group (n=40), compared to CSRG-NF (n=72) patients. A trend was also seen for DLCO SB (p=0.063). P-values determined by Wilcoxon rank-sum test. **(C)** No significant correlations of fatigue were found in the UofA cohort. **(D)** FACIT fatigue scores were found to significantly correlate with all lung function measures and disease damage accrual (SCTC-DI), using Spearman’s rho, Bonferroni corrected.

Most lung function indices were within normal ranges in the UofA cohort with 86.3% of patients having % predicted values over 70% in FVC, TLC and DLCO SB. Median % predicted DLCO SB values were significantly lower (73vs 89, p=0.028) in SSc-F patients, % predicted TLC was also markedly lower, though not significant (85 vs 93.5, p=0.105) (**Supplementary Table 4**, **Figure 2A**). Actual DLCO SB values were lower in SSc-F patients (15.37 vs 19.18, p=0.046), with a trend evident for lower TLC values (4.78 vs 5.01, p=0.077). When controlling for age, disease duration and disease damage accrual, no significant correlates were observed for fatigue (FACIT) in the UofA cohort (**Figure 2C**, **Supplementary Figure 4A**).

In the CSRG cohort, 89.6% of patients had TLC and FVC % predicted values over 70%, however only 65% had DLCO SB values over 70%. Median values for FVC (% Pred) (92 vs 98.5, p=0.039) and TLC (% Pred) (92 vs 99, p=0.034) were significantly lower in CSRG-F patients with a trend was also evident for decreased DLCO SB (% Pred) (71 vs 76, p=0.063) (**Supplementary Table 6**, **Figure 2B**). TLC values were also lower in SSc-F patients (4.32 vs 5.02, p=0.026). When controlling for age, disease duration and damage accrual, FVC (% Pred) was found to moderately correlate with fatigue (FACIT) (rho:0.362, p=0.004). DLCO SB (% Pred) (rho:0.307, p=0.036) and disease damage accrual (SCTC-DI) (rho: -0.300, p=0.046) also weakly correlated with fatigue (**Figure 2D**, **Supplementary Figure 4**).

### 3.5 Microvascular changes associated with pulmonary involvement are more common in SSc patients with severe fatigue

**Figure 3:**
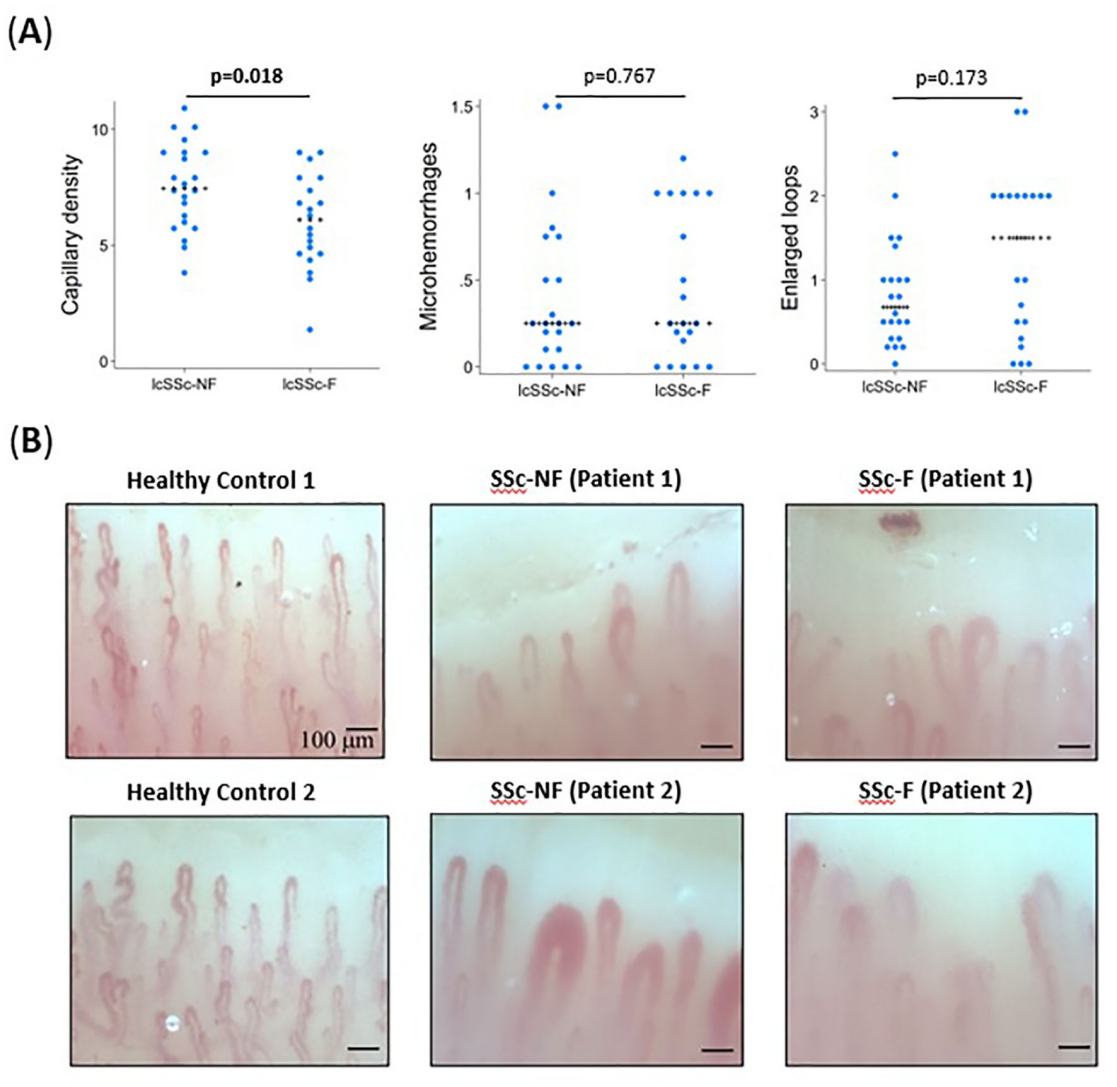
Nailfold capillaroscopy findings associated with severe fatigue in systemic sclerosis patients. **(A)** In the UofA cohort (n=42), SSc-F patients were found to have significantly lower capillary density (p=0.018) than SSc-NF patients. Though not significant, the number of enlarged loops was also higher in SSc-F patients. All values are the average number of observations per digit across eight digits. P-values determined by Wilcoxon rank-sum test. **(B)** Representative images of healthy control, SSc-NF and SSc-F patients. Abnormal features including the presence of dilated capillaries, microhemorrhages, and decreased capillary density are evident in both SSc patient groups.

Patients in the UofA cohort were examined using nailfold video capillaroscopy (NVC), and as previously noted, all patients had a SSc specific NVC pattern (**Figure 3**). We utilized our NVC data to determine if there were changes present in the SSc-F group that may reflect systemic vascular remodeling. Notably, capillary density was found to be significantly lower in the SSc-F group (6.1 vs 7.45, p=0.018) (**Figure 3**, **Supplementary Table 3**). Capillary dropout, giant loops, enlarged loops and microhemorrhages per digit were however similar between SSc-F and SSc-NF patients. (**Figure 3**, **Supplementary Table 4**).

### 3.6 Gal-9 is elevated in SSc patients with severe fatigue and diminished lung function

**Figure 4:**
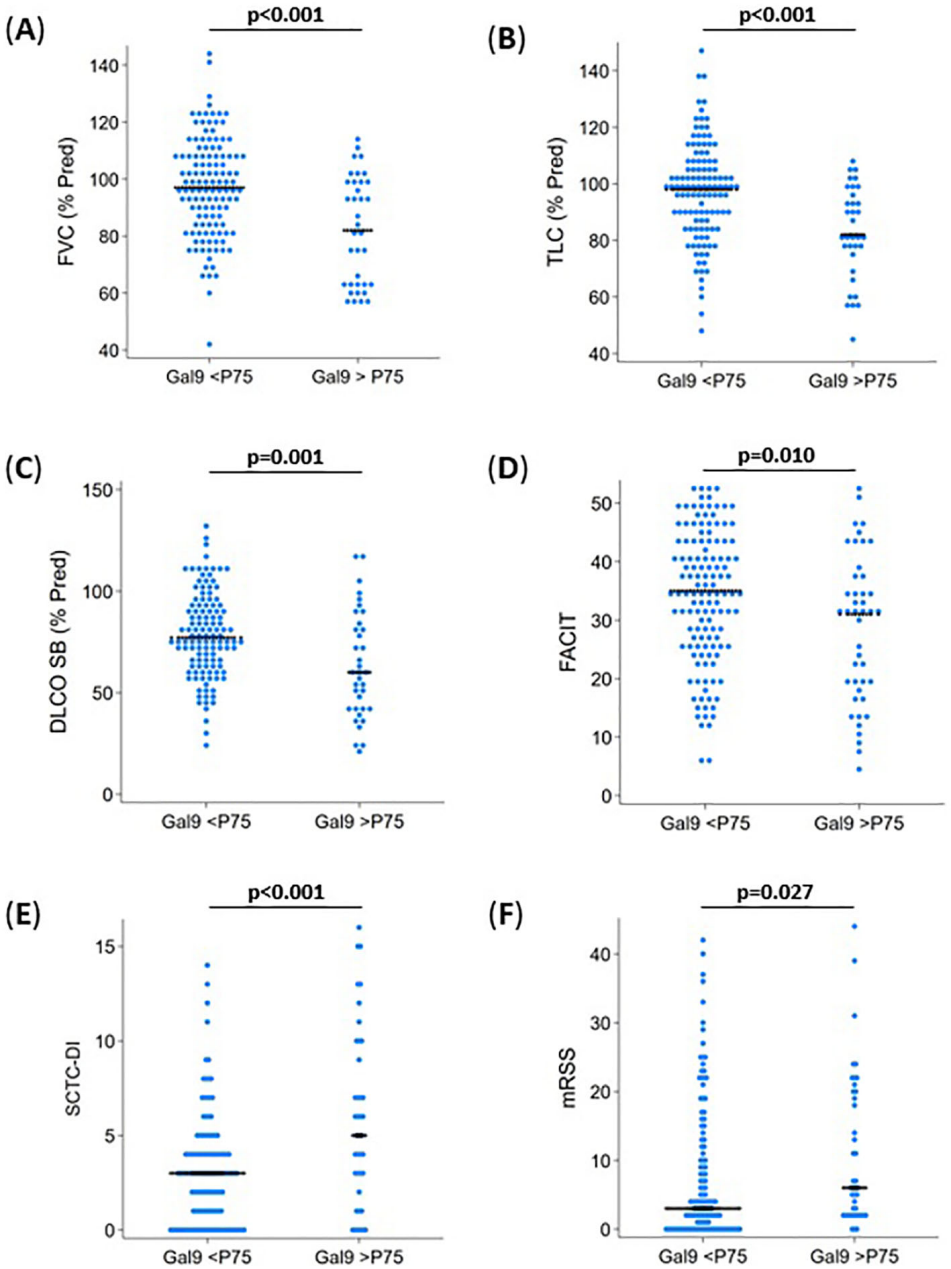
Galectin-9 and its association with fatigue and disease measures in systemic sclerosis. Patients from the UofA and CSRG groups were combined (n=174) and the 75th percentile (P75) Gal-9 level used to separate patients, to determine associations with disease measures. **(A)** FVC (% Pred) was lower in patients with >P75 Gal-9 levels (p<0.001). **(B)** TLCC (% Pred) was lower in patients with >P75 Gal-9 levels (p<0.001). **(C)** DLCO SB (% Pred) was lower in patients with >P75 Gal-9 levels (p=0.001). **(D)** Fatigue (FACIT) were lower in patients with >P75 Gal-9 levels (p=0.010), indicating a greater level of fatigue. **(E)** Disease damage accrual (SCTC-DI) was higher in patients with >P75 Gal-9 levels (p<0.001). **(F)** Skin involvement was higher in patients with >P75 Gal-9 levels (p<0.027). p-values determined by Wilcoxon rank-sum test.

In the UofA SSc cohort, Gal-9 levels were elevated in SSc-F patients (2303.91 pg/ml) when compared to SSc-NF patients (1654.10 pg/ml) (p=0.042) (**Supplementary Table 4**). Similarly, serum Gal-9 levels in the CSRG cohort also differed significantly between CSRG-F (4834.47 pg/ml) and CSRG-NF (3594.01pg/ml) patients (p=0.046) (**Supplementary Table 6**). We grouped both cohorts and determined the 75th percentile for Gal-9 (5583.394), we used this as a cut-off and split the patients into a group below the 75th percentile (<P75) and one above or equal to the 75th percentile (>P75). We then determined the association between Gal-9 levels and changes associated with lung fibrosis and disease-associated damage accrual. We found that SSc patients with Gal-9 levels >P75, had lower FVC (% Pred) (p<0.001), TLC (% Pred) (p<0.001), and DLCO SB (% Pred) (p=0.001) (**Figures 4A–C**). These patients also had elevated skin involvement (mRSS) (p=0.027), disease accrual (SCTC-DI) (p<0.001), and lower FACIT fatigue scores (p=0.010) (**Figures 4D–F**) – highlighting the potential relationship between elevated Gal-9, SSc-associated damage indices, and fatigue.

### 3.7 Cytokines associated with fibrosis and vascular remodeling are elevated in SSc-F patients, and correlate with Gal-9

We screened plasma samples from the UofA cohort for changes in cytokines, chemokines, and growth factors associated with fibrosis (e.g. interleukin 6, C-reactive protein), and vascular changes (e.g. IL-16, VEGF, LT-α) (**Supplementary Figure 5**, **Supplementary Table 7**). Elevations in LT-α (p=0.008), IL-16 (p=0.006), PIGF (p=0.015), and VEGF (p=0.011) were observed in SSc-F patients (**Supplementary Figure 5**). As Gal-9 has been linked with various cytokines that promote, severe fatigue and cognitive dysfunction in other forms of inflammatory ME/CFS, we aimed to further determine which cytokines positively correlated with Gal-9 values in the UofA cohort. CRP (rho:0.449, p=0.009), LTα (rho:0.487, p=0.004), and IL-6 (rho: 0.400, p=0.021), all of which are associated with lung fibrosis were found to correlate with Gal9 levels (**Supplementary Figures 5**, **6**).

## 4 Discussion

Although fatigue is a common complaint in many systemic autoimmune rheumatic disease (27) patients, it is rarely given appropriate (if any) attention in the clinical setting (2). The severity of fatigue and its association with symptoms such as PEM, non-refreshing sleep, and cognitive failure, as found in ME/CFS, is bothersome – particularly when there is no active inflammatory component present in early SSc patients that requires augmented immunomodulation (28). We have previously shown that subgroups of SARD patients have symptoms severe enough to meet the diagnostic criteria for ME/CFS (6, 7). In our current study, we have shown that severe fatigue outweighed all other variables, including factors associated with SSc-related damage accrual in its association with disability scores (HAQ-DI). Notably, nearly a third of the SSc patients we identified that suffered from severe fatigue were unemployed and/or were on disability welfare, a finding that underscores the impact of severe fatigue on SSc patients’, physical, social, mental and financial well-being.

Our study explores the hypothesis that severe fatigue in early SSc patients, may be indicative of increased fibrotic disease. Direct comparison of fatigued and non-fatigued patients did not find any differences in disease indicators, such as, skin involvement, disease damage accrual, or disease subtype. Subclinical findings however, indicated that pulmonary function test (PFT) values were diminished in patients with severe fatigue from both cohorts. As this was a cross-sectional study, we did not assess longitudinal changes in lung function (29).

Microvascular damage is an early event in the pathogenesis of SSc, with digital vasculopathy and the preceding manifestation of Raynaud’s phenomenon being evident early in disease progression (30). NVC is known to predict visceral involvement – particularly SSc-PAH (31), and SSc-ILD (32, 33), which stems from progressive and obliterative vascular remodeling in the pulmonary circulation. In particular, SSc patients with a loss in capillary density were shown to present with worse FVC and DLCO values (32). Our findings are in keeping with this, as SSc-F patients were found to have decreased nailfold capillary density. Dysregulated angiogenesis and endothelial dysfunction play a significant role in the progression of vascular injury and fibrosis in SSc (30). These results may also suggest that SSc patients (in general) may harbor defects in their oxygen transport cascade; with fatigued patients carrying a higher risk of this as they may also have added reduced lung diffusion capacity/total lung capacity and mitochondrial dysfunction, as we have previously suggested (7). The mechanism(s) related to this are largely unexplored.

Our results linking diminished lung function and vasculopathy with severe fatigue in patients with SSc, inspired us to explore the possibility of Gal-9 elevation as a potential indicator of disease-associated damage accrual in both the UofA and CSRG cohorts. Gal-9 has been suggested to be a promoter of vascular remodeling (34), and is associated with increased disease severity in SSc patients (16, 17). We found that elevated Gal-9 levels in SSc patients with severe fatigue, was associated with reduced lung function (DLCO SB, FVC, TLC), and increased disease indicators (SCTC-DI, mRSS). TLC is seldom used in the prediction of SSc disease activity, although recently, a large French cohort study suggested that TLC is an earlier predictive marker for ILD and lung fibrosis than FVC (35).

Soluble Gal-9 acts as a damage-associated molecular pattern (PAMP) and is associated with cytokine release syndrome, and immune cell activation (36, 37). Hence, the elevation of Gal-9 in SSc patients, could exacerbate vascular remodeling promoted by immune cells, which is central to the pathogenesis of SSc (34, 38–40). Gal-9 is also a known promoter of mitochondrial dysfunction (41, 42), which we have previously shown in SSc (7). Numerous cytokines have been linked to SSc disease outcomes (10, 11, 30, 43–49), though findings are often discordant (13, 30, 43, 49), suggesting that multiple cytokines would likely need to be evaluated to discern patients which may be at a higher risk for pulmonary and vascular involvement. Our finding that Gal-9 correlates with some of these cytokines (IL-16, VEGF, LTα), as well as with lung function parameters and disease accrual, suggests that it may be a good candidate for patient stratification. Although it is tempting to speculate that Gal-9 dysregulation may directly contribute to defects related to oxygen consumption (41, 50), future studies assessing this are required.

There are several limitations to this study. First, the UofA cohort was of single-centered cross-sectional design, with a relatively small sample size without serial follow-up measurements. Larger sized multi-centered studies are currently underway, as changes in lung function may require longer periods (29). Second, our study focused on Gal-9 as an indicator of hypoxic vascular remodeling due to its link with increased organ fibrosis and mortality in SSc patients. Other indicators linked with the biology of fibrosis in SSc (e.g. interferon score) may be associated with Gal-9 as suggested in other similar disease states (51), and may thus be the focus of future research related to SSc. Third, SSc is more prevalent in females, and in both our cohorts they accounted for over 8o% of participants. Thus, sex-based analyses were not conducted. Lastly, though race was captured, both cohorts were comprised of mostly white study subjects (>80%), which may make our observations less generalizable to other diverse populations.

**Figure 5:**
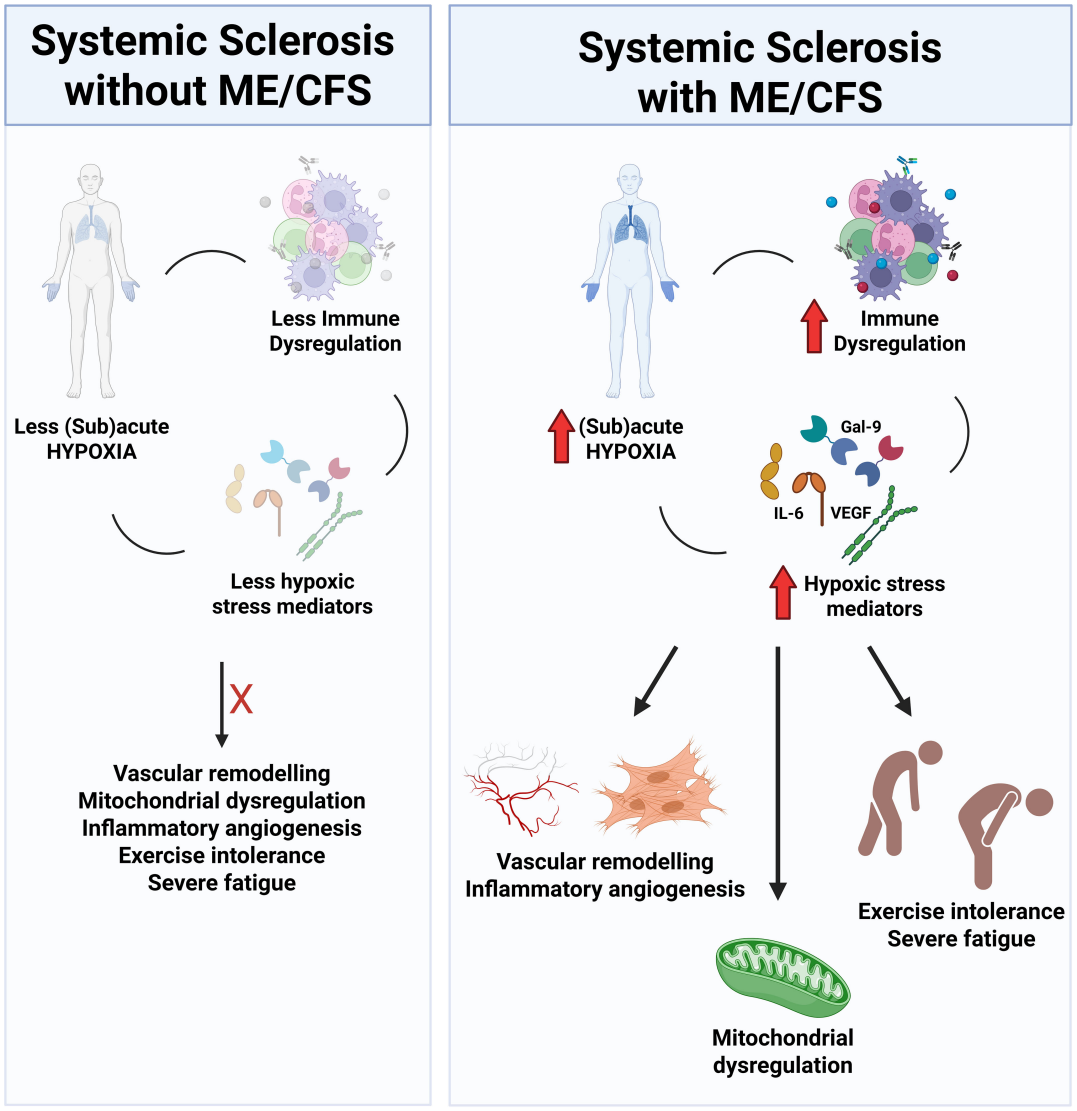
Schematic representation of factors associated with ME/CFS in SSc. Levels of subacute hypoxia (reduced capillary density, reduced lung function parameters) and hypoxic stress mediators (ie. Gal-9, IL-6, VEGF) are elevated in SSc-CFS patients, which culminate in vascular remodeling, inflammatory angiogenesis, mitochondrial dysregulation and the development of reduced exercise tolerance and sever fatigue.

In conclusion, we propose that physicians should determine the presence of severe fatigue in all SSc patients, as these symptoms may possibly reflect sub-clinical disease associated complications even early in their disease journey. We suspect that patients with SSc (in general) may be at increased risk for developing ME/CFS-like symptoms, which may reflect defects in their oxygen transport cascade (e.g. ILD and mitochondrial dysfunction) (**Figure 5**). Further to this, elevated Gal-9 values may prove helpful in the stratification of these patients. Understanding the mechanisms governing severe fatigue in SSc patients may be key to the reduction of poor outcomes and improved quality of life in SSc and other rheumatic disease patients.

## Data Availability

Data collected for this study, including individual participant data, will not be made available to others to minimize breeches in confidentiality as per our REB requirements. Requests to access the datasets should be directed to mosman@ualberta.ca.
