# Supplementary material for: Severe fatigue is associated with diminished lung function and elevated Galectin-9 levels in early systemic sclerosis
Source: Front Immunol. 2025 Sep 5;16:1655414. doi: 10.3389/fimmu.2025.1655414 (PMC12447584; doi:10.3389/fimmu.2025.1655414)
Supplement: Supplementary file 1 [file DataSheet1.docx]

Supplementary Data

**Supplementary Table 1. Mitogen^Dx^ antibody panel**

|  | **Systemic Sclerosis Panel** |
| --- | --- |
| **Antibodies**  (MitogenDx laboratories, Calgary, Alberta) | Anti-CENP A + B, Topo-I/Scl-70, RNA polymerase III, fibrillarin, Th/To/hPOP1, Ku, PDGFR, Ro52/TRIM21, PM21, PM/Scl-75, PM/Scl-100, Nor90/Hubf, Jo-1, Mi2, Mi2-α, Mi2β, MDA5, NXP2, TIF1γ, PL7, PL12, SRP, EJ, OJ |

**Questionnaires**

All questionnaires listed below, are validated, reliable tools, which are freely available for use.

All patients completed the DePaul Questionnaires (DSQ-2), a self-reported validated measure to assess the presence of ME/CFS symptoms such as fatigue, post-exertional malaise, sleep, pain, and neurological/cognitive impairments, autonomic, neuroendocrine, and immune-related symptoms. DePaul questionnaire contains questions with the purpose of determining the frequency and severity of ME/CFS symptoms. Frequency is rated on a scale of 0 to 4, with 0=none of the time, 1= little of the time, 2= about half of the time, 3= most of the time, 4= all of the time over the past 6 months; and severity rated again on a scale of 0 to 4, with 0= no symptoms, 1= mild, 2= moderate, 3=severe and 4 very severe. The patients considered as having ME/CFS needed to meet ≥ 5 out of the 6 diagnostic criteria.^1^

The MFI is a self-reported 20-item scale designed to evaluate five dimensions of fatigue: general fatigue, physical fatigue, reduced motivation, reduced activity, and mental fatigue. Items are scored 1–5, with 10 [fatigue-] positively phrased items reverse scored (items 2, 5, 9, 10, 13, 14, 16, 17, 18, 19). In the final score, higher scores represent more acute levels of fatigue.^2^

The Functional Assessment of Chronic Illness Therapy (FACIT), a self-reported 13-item questionnaire that measures the level of fatigue during usual daily activities over the past 7 days. Items are measure on a four-point Likert scale (0= not at all, 1= a little bit, 2= somewhat, 3= quite a bit and 4= very much) except items 7 and 8 which are reversed scored. Score ranges between 0-52 with a score less than 30 indicates serve fatigue and the higher the score, the better the quality of life.^3^

For assessing self-reported health-related quality of life in patients, we used The Short Form (36) Health Survey questionnaire, with scores ranging from 0-to 100. Lower scores in the following categories: vitality, physical functioning, body pain, the general perception of health, physical role functioning, emotional role functioning, social role functioning, and mental health, indicated more fatigue and disability. These eight domains can be summarized into a Physical Component Score (PCS) and a Mental Component Score (MCS). A final score for each domain is provided between 0-100, with 0 being the worst possible health and 100 the best possible health. The score is standardized against normative population data, where the mean score ±SD of 50 ± 10 is the population normative score.^4^

The Hospital Anxiety and Depression Scale (HADS) questionnaire, is a 14-item self-reported screening scale to identify the presence of anxiety and depression in patients was used in our survey. This tool contains 7 anxiety-related and 7 depression-related items, with scores of each ranging from 0-3, and with a total score of 11 or above indicative of abnormal results.^5^

Sleep disturbances were assessed using The Pittsburgh Sleep Quality Index (PSQI), which is a self-reported questionnaire to evaluate a wide variety of factors relating to sleep quality. These include estimates of sleep duration, latency, frequency, and severity of specific sleep-related problems. This tool measure seven domains (subjective sleep quality, sleep latency, sleep duration, habitual sleep efficiency, sleep disturbances, use of sleep medication, and daytime dysfunction) over the past month, with answers based on a 0-3 (0= not during the past month, 1= less than once a week, 2= once or twice a week and 3= three or more times week) scale. A global score of 5 or greater indicates poorer sleep quality.^6^

**Supplementary Table 2:** Questionnaires used for assessing patient symptoms

| **Abbreviation** | **Questionnaire** | **Reference** |
| --- | --- | --- |
| DSQ-2 | DePaul Symptom Questionnaire | 1 |
| MFI | Multidimensional Fatigue Inventory | 2 |
| FACIT | The Functional Assessment of Chronic Illness Therapy | 3 |
| SF36 | Short Form - 36 | 4 |
| HADS Anxiety | Hospital Anxiety and Depression Scale | 5 |
| HADS Depression | Hospital Anxiety and Depression Scale | 5 |
| PSQI | Pittsburgh Sleep Quality Index | 6 |
| CFQ | Cognitive Failure Questionnaire | 7 |
| HAQ-DI | Health Assessment Questionnaire – Disability Index | 8 |

The Cognitive Failure questionnaire consists of 25 self-reported items to analyze the frequency of cognitive failure. Factors assessed included absent-mindedness, slips and errors, memory, and motor functioning in everyday life. The scoring is from 0-100, with scores of ≥43 is indicative of cognitive impairment.^7^

The Health Assessment Questionnaire - Disability Index (HAQ-DI) assesses a patient's level of functional ability. It contains 20 questions in eight categories of functioning which represent a comprehensive set of functional activities – dressing, rising, eating, walking, hygiene, reach, grip, and usual activities. The patient's responses are made on a scale from zero (no disability) to three (completely disabled).^8^


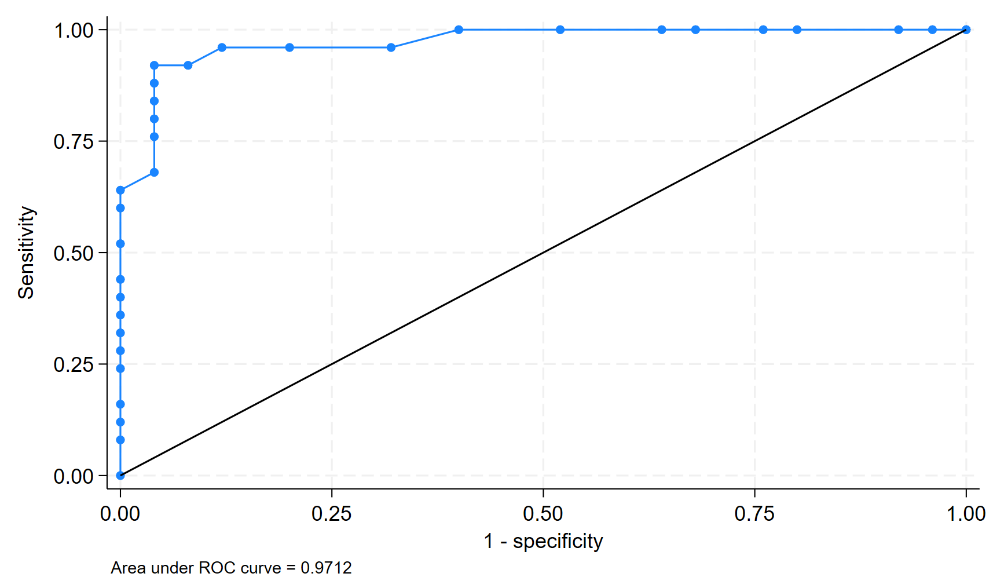


**Supplementary Figure 1:** The receiver operating characteristic (ROC) curve of comparison between FACIT fatigue scores and ME/CFS classification based on the international criteria. The receiver operating characteristic (ROC) curve, comparing ME/CFS classification to FACIT fatigue scores had an area under the curve of 0.9712. ROC analysis for the classification of patients by the <30 FACIT score cut-off (as an indicator of severe fatigue), had an area under the curve of 0.9000 (CI: 0.816;0.983. FACIT (<30) had good accuracy to ME/CFS classification with good accuracy, with 84.0% sensitivity, 96% specificity and an accuracy of 90%.

Validation of serum and plasma samples for Galectin 9 ELISA.

The Galectin-9 ELISA kit used in this study (DY2045, R&D Systems) is suitable for use in both serum and plasma. Our group has utilised this kit with plasma on multiple occasions, particularly in COVID-19 patients^9,10^. Unfortunately, we were not able to get plasma samples for the CSRG cohort, and thus used serum. This kit has however been used with serum for both SSc^11^ and COVID-19^12^. We compared Gal-9 expression between serum and plasma samples in six SSc patients from the UofA cohort. We found that the mean, standard error and 95% confidence intervals for Galectin-9 between serum and plasma was similar, with paired (Supplementary Figure 2) t-test analysis showing no significant differences (p=0.6173).


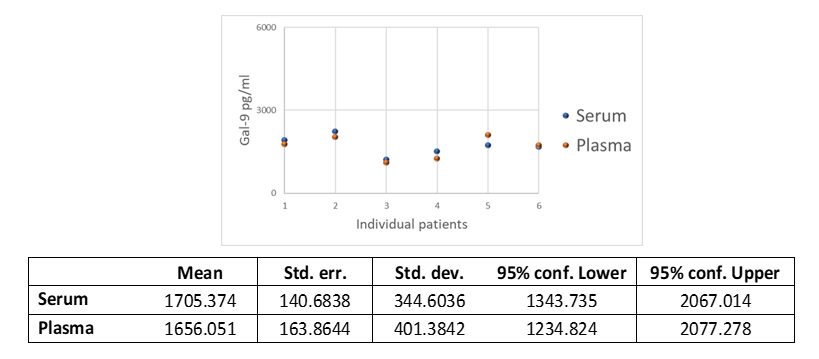


**Supplementary Figure 2:** Comparison between Gal-9 levels in individual patients form both serum (blue) and plasma (orange). Paired t-test analysis comparing serum and plasma samples used for the Gal-9 ELISA from six patients, found no significant difference p=0.6173).

**Supplementary Table 3. Patient characteristics of the UofA cohort**

|  | **Study Cohort (UofA)** | | |
| --- | --- | --- | --- |
|  | **SSc-NF**  **(n=26)** | **SSc-F**  **(n=25)** | **p-value** |
|  | *Count (%)* | *Count (%)* |  |
| Sex (F) | 21/26 (80.7) | 20/25 (80.0) | 0.945 |
| Disease type (dcSSc) | 2/26 (7.69) | 3/25 (12.0) | 0.959 |
| Sleep disturbances | 16/26 (61.5) | 22/25 (88.0) | **0.030** |
| Fibromyalgia | 1/26 (3.8) | 14/25 (56.0) | **<0.001** |
| Cognitive failure | 2/25 (8.0) | 11/25 (44.0) | **0.004** |
| Depression | 0/24 (0) | 6/25 (24.0) | **0.010** |
| Anxiety | 1/24 (0) | 6/25 (24.0) | **0.047** |
| Digital Ulcers | 4/26 (15.3) | 7/24 (29.1) | 0.240 |
| Puffy Fingers | 20/26 (76.9) | 16/24 (66.6) | 0.420 |
| Inflammatory Arthritis | 5/26 (19.2) | 9/24 (37.5) | 0.151 |
| PAH | 3/26 (11.5) | 2/25 (8.0) | 0.671 |
| ILD | 9/26 (34.6) | 9/25 (36.0) | 0.918 |
| Smoking History (Y) | 15/24 (62.5) | 11/24 (45.8) | 0.247 |
| Medications  *None*  *Vasodilators*  *Immune Regulators*  *Vasodilators & Immune Reg* | 3/25(12.0)  4/25 (16.0)  9/25 (36.0)  9/25 (36.0) | 1/25 (4.0)  6/25 (24.0)  6/25 (24.0)  12/25 (48.0) | 0.488 |
| Antibodies  *ANA* (*SSc specific Abs negative)*  *Anti-Topoisomerase I*  *Anti-RNA polymerase III (RNPIII)*  *Anti-Centromere (ACA)*  *ACA + RNPIII* | 5/24  8/24  0/24  11/24  0/24 | 10/22  3/22  1/22  7/22  1/22 | 0.149 |
|  | *Median (IQR)* | *Median (IQR)* |  |
| Age | 58 (46;63) | 49 (42;62) | 0.479 |
| Disease Duration | 1.8 (0.7;3) | 1 (0;3) | 0.816 |
| SF36 – PCS | 53.65 (48.8;56.5) | 29.4 (26.7;37.1) | **<0.001** |
| SF36 - MCS | 53.6 (49.8;57.9) | 37.75 (30.6;44.1) | **<0.001** |
| MFI | 41 (34;50) | 72 (64;78) | **<0.001** |
| FACIT | 45 (41;48) | 22 (16;27) | **<0.001** |
| HAQ DI | 0 (0;0.18) | 0.75 (0.62;0.87) | **<0.001** |
| CRP (mg/L) | 2.4 (0.6;5.8) | 2.9 (0.8;6.8) | 0.299 |
| BMI | 27.14 (23.3;29.7) | 26.05 (21.8;32.1) | 0.664 |
| mRSS | 0 (0;3) | 0 (0;4) | 0.579 |
| SCTC-DI | 1 (0;3) | 3 (1;5) | 0.050 |

Abbreviations

F: female; dcSSc: diffuse systemic sclerosis; SSc-F: systemic sclerosis with severe fatigue; SSc-NF: systemic sclerosis without severe fatigue; BMI: body mass index; MFI: multidimensional fatigue inventory; SF-36: 36-item short form health survey, PCS: physical component score, MCS: mental component score; HAQ DI: health assessment questionnaire disability index; CRP: C-reactive protein; mRSS: modified Rodnan skin score; SCTC-DI: scleroderma clinical trials consortium damage index; ANA: antinuclear antibody.

**Supplementary Table 4. Further demographics and patient characteristics of SSc-NF and SSc-F patients, UofA cohort**

|  | **Study cohort (UofA)** | | |
| --- | --- | --- | --- |
|  | **SSc-NF**  **(n=26)** | **SSc-F**  **(n=25)** | **p-value** |
|  | *Count (percentage)* | *Count (percentage)* |  |
| Employment  *On Disability*  *Homemaker*  *Retired*  *Unemployed*  *Part-Time*  *Full-Time* | 0/24 (0)  0/24 (0)  6/24 (25.0)  0/24 (0)  3/24 (12.5)  15/24 (62.5) | 8/25 (32.0)  1/25 (4.0)  7/25 (28.0)  1/25 (4.0)  3/25 (12.0)  5/25 (20.0) | **0.010** |
| ATTG | 0/24 (0) | 0/25 (0) | . |
| DAT | 0/24 (0) | 0/25 (0) | . |
| Skin Involvement (mRSS >0) | 10/26 (38.4) | 11/24 (45.8) | 0.598 |
| Lung Involvement | 10/26 (38.4) | 11/25 (44.0) | 0.688 |
| Heart Involvement | 2/26 (7.6) | 2/25 (8.0) | 0.967 |
| GI Tract Involvement | 22/26 (84.6) | 19/25 (76.0) | 0.439 |
| Kidney Involvement | 0/26 (0) | 1/25 (4.0) | 0.303 |
|  | *Median (IQR)* | *Median (IQR)* |  |
| PSQI | 5.5 (3;8) | 12 (8;15) | **<0.001** |
| WPI | 1 (0;4) | 7 (5;12) | **<0.001** |
| SSS | 3 (2;3) | 7 (6;8) | **<0.001** |
| TSH (mU/L) | 2.15 (1.36;2.88) | 2.52 (1.8;3.6) | 0.295 |
| Vitamin B12 (pmol/L) | 323 (269;367) | 355 (277;487.5) | 0.345 |
| Hemoglobin A1c (%) | 5.45 (5.1; 5.7) | 5.5 (5.05;5.75) | 0.888 |
| Galectin-9 | 1654.10 (1127.32;2648.77) | 2303.91 (1763.29;4464.93) | **0.042** |
| FVC (% Pred) | 97 (86;110) | 91 (74;104) | 0.153 |
| DLCO SB (% Pred) | 89 (77;97) | 73 (61;84) | **0.028** |
| TLC (% Pred) | 93.5 (90;105) | 85 (74;100) | 0.105 |
| SpO2 (%) | 97.5 (95;99) | 97 (96;98) | 0.728 |
| FVC (L) | 3.32 (2.79;3.94) | 3.02 (2.64;3.7) | 0.161 |
| DLCO SB (ml/(min*mmHg)) | 19.18 (16.26;22.85) | 15.37 (12.5;20.4) | **0.046** |
| TLC (L) | 5.015 (4.67;5.65) | 4.78 (4.01;5.25) | 0.077 |
| Nailfold Capillaroscopy  *Microhemorrhages*  *Giants per digit*  *Enlarged per digit*  *Capillary density*  *Capillary dropout* | 0.25 (0.1;0.75)  0.4 (0.25;1)  0.675 (0.25;1)  7.45 (5.9;9)  1 (1;1) | 0.25 (0.06;1)  0.5 (0.25;2)  1.5 (0.37;2)  6.1 (4.55;7.7)  1 (1;2) | 0.767  0.368  0.173  **0.018**  0.291 |

Abbreviations

SSc-F: systemic sclerosis with ME/CFS; SSc-NF: scleroderma without ME/CFS; ATTG: Anti-tissue transglutaminase antibodies; DAT: direct antiglobin test; GI tract: gastrointestinal tract; BMI: body mass index; MFI: multidimensional fatigue inventory; FACIT: functional assessment of chronic illness therapy; PSQI: Pittsburgh sleep quality index; WPI: widespread pain index; SSS: symptom severity scale; SF-36: 36-item short form health survey, PCS: physical component score, MCS: mental component score; TSH: Thyroid stimulating hormone.

**Supplementary Table 5. Patient characteristics of the CSRG cohort**

|  | **Comparison Cohort (CSRG)** | | |
| --- | --- | --- | --- |
|  | **CSRG-NF**  **(n=81)** | **CSRG-F**  **(n=45)** | **p-value** |
|  | Count (%) | Count (%) |  |
| Sex (F) | 62/81 (76.4) | 42/45 (93.3) | **0.017** |
| Disease type (dcSSc) | 26/81 (32.0) | 19/45 (42.2) | 0.256 |
| Digital Ulcers | 7/81 (8.6) | 3/45 (6.6) | 0.694 |
| Puffy Fingers | 52/81 (64.1) | 23/45 (51.1) | 0.152 |
| PAH | 6/61 (9.8) | 4/35 (11.4) | 0.899 |
| ILD | 22/81 (27.1) | 14/45 (31.1) | 0.638 |
| Smoking History (Y) | 45/81 (55.5) | 34/45 (75.5) | 0.111 |
| Medications  *None*  *Vasodilators*  *Immune Regulators*  *Vasodilators & Immune Reg* | 29/81 (35.8)  26/81 (32.0)  11/81 (13.5)  15/81 (18.5) | 10/45 (22.2)  10/45 (22.2)  10/45 (22.2)  7/45 (15.5) | 0.298 |
|  | *Median (IQR)* | *Median (IQR)* |  |
| Age ^13^ | 54 (47; 63) | 53 (46; 60) | 0.490 |
| Disease Duration ^13^ | 2.47 (1.48; 3.36) | 2.38 (1.23; 3.66) | 0.816 |
| SF36 – PCS | 44.5 (37.4; 50.4) | 32.0 (25.6; 37.5) | **<0.001** |
| SF36 - MCS | 52.1 (42.2; 57.4) | 39.5 (32; 50.1) | **<0.001** |
| FACIT | 38 (34; 44) | 20 (15; 25) | **<0.001** |
| HAQ DI | 0.375 (0;1) | 1.37 (0.5; 1.75) | **<0.001** |
| CRP (mg/L) | 3 (1.4; 7.4) | 6 (2.1; 10.0) | **0.043** |
| BMI | 24.7 (22.2;28.2) | 26.3 (22.8;32.2) | 0.126 |
| mRSS | 5.5 (2; 13.5) | 7 (3; 19) | 0.271 |
| SCTC-DI | 3 (1;5) | 4 (3;6) | 0.136 |

Abbreviations

F: female; dcSSc: diffuse systemic sclerosis; CSRG-F: CSRG cohort, systemic sclerosis with severe fatigue; CSRG-NF: CSRG cohort, systemic sclerosis without severe fatigue; BMI: body mass index; FACIT: functional assessment of chronic illness therapy; SF-36: 36-item short form health survey, PCS: physical component score, MCS: mental component score; HAQ DI: health assessment questionnaire disability index; CRP: C-reactive protein; mRSS: modified Rodnan skin score; SCTC-DI: scleroderma clinical trials consortium damage index.

**Supplementary Table 6. Further demographics and patient characteristics of SSc-NF and SSc-F patients, CSRG cohort**

|  | **CSRG-NF** | **CSRG-F** | **p-value** |
| --- | --- | --- | --- |
|  | Median (IQR) | Median (IQR) |  |
| Galectin 9 | 3594.008 (2602.50; 5603.74) | 4834.478 (3271.75; 6475.59) | **0.046** |
| FVC (% Pred) | 98.5 (85;108) | 92 (76.5;103.5) | **0.039** |
| DLCO SB (% Pred) | 76 (61;91) | 71 (51;78) | 0.063 |
| TLC (% Pred) | 99 (88;109) | 92 (77;102) | **0.034** |
| FVC (L) | 3.115 (2.63; 3.68) | 2.72 (2.20;3.43) | 0.107 |
| DLCO SB (ml/(min*mmHg)) | 17.06 (13.1;20.86) | 14.6 (12.4;19.78) | 0.206 |
| TLC (L) | 5.02 (4.14;5.72) | 4.32 (3.7;5.41) | **0.026** |

**Supplementary Table 7. Cytokine and protein markers associated with severe fatigue in SSc, UofA cohort**

|  | **SSc-NF (n=17)** | **SSc-F (n=20)** |  |
| --- | --- | --- | --- |
|  | *Count (percentage)* | *Count (percentage)* | *p-value* |
| LTα | 1.74 (1.34; 2.10) | 2.44 (1.90; 2.86) | **0.008** |
| CRP | 2.36^e+08^ (3.72^e+07^; 9.00^e+08^) | 6.13^e+08^ (1.69^e+08^; 1.13^e+09^) | 0.140 |
| IL-6 | 0.75 (0.44; 1.16) | 0.94 (0.60; 1.79) | 0.211 |
| IL-15 | 1.68 (1.52; 2.43) | 2.01 (1.39;2.71) | 0.394 |
| IL-16 | 102.69 (79.38; 141.0) | 167.17 (127.8; 206.8) | **0.006** |
| IP10 | 596.55 (352.9; 892.6) | 680.48 (491.5; 1174.8) | 0.497 |
| MIP-1α | 25.75 (20.42; 30.99) | 27.47 (22.70; 38.05) | 0.211 |
| PlGF | 146.79 (130.26, 172.15) | 227.10 (159.4; 264.2) | **0.015** |
| VEGF | 16.94 (11.33; 24.86) | 25.61 (19.02; 33.18) | **0.011** |


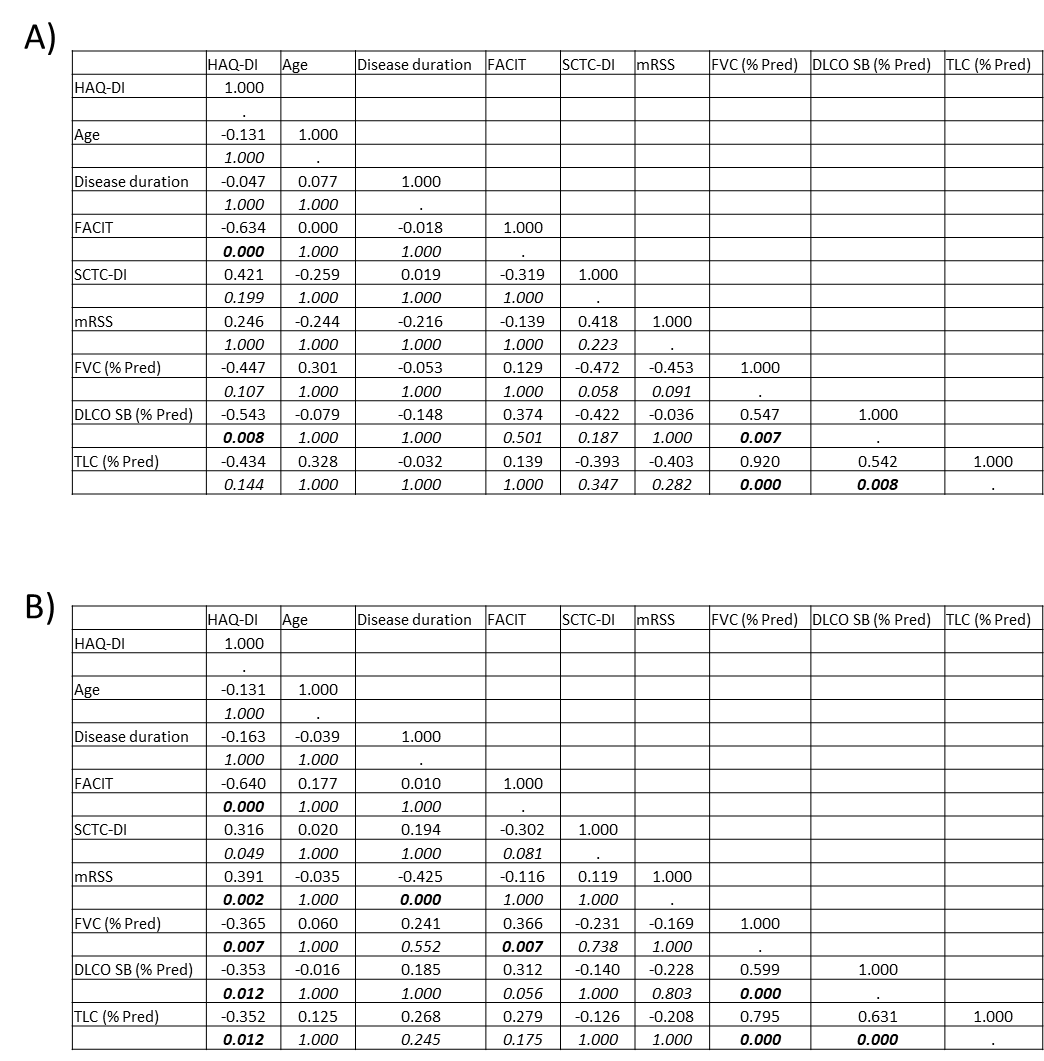


**Supplementary Figure 3. Correlates of disability (HAQ-DI) in SSc.** Bonferroni corrected Spearman correlations showing the relationship between disabilty, and fatigue and disease associated parameters in SSc patients in both the UofA **(a)** and CSRG **(b)** cohorts.


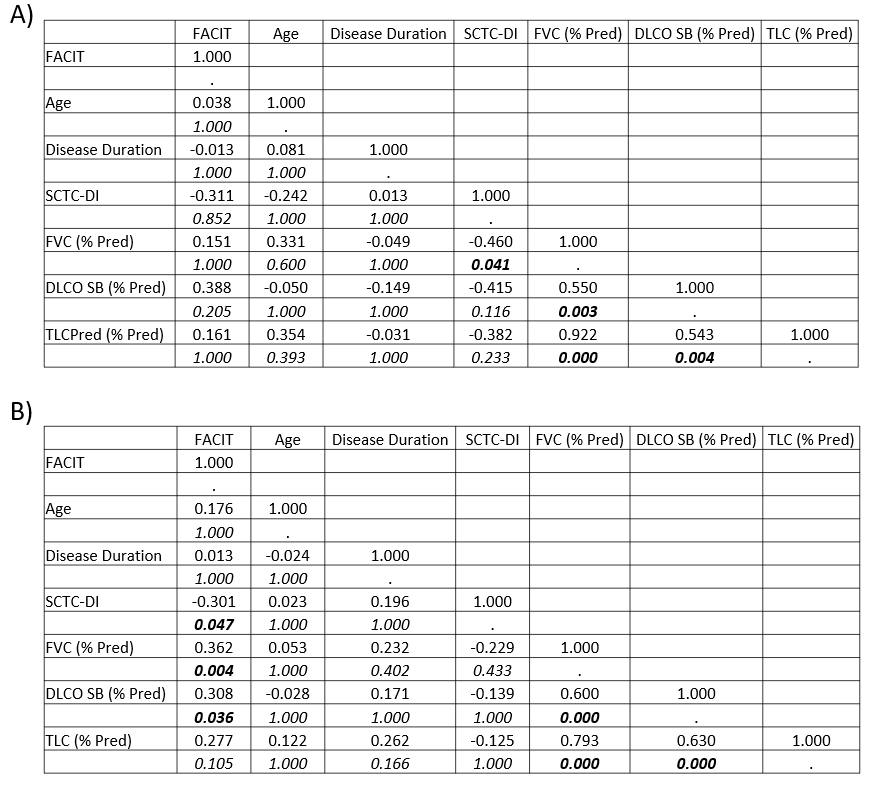


**Supplementary Figure 4. Correlates of fatigue (FACIT).** Bonferroni corrected Spearman correlations showing the relationship between FACIT scores and disease associated parameters in both the UofA and CSRG cohorts. No significant associations were seen in the UofA cohort (**a,b**)**.** In the CSRG cohort, both DLCO SB and FVC were significantly correlated with fatigue, as was disease activity (SCTC-DI) (**c,d**). Striped bars indicate negative correlations.


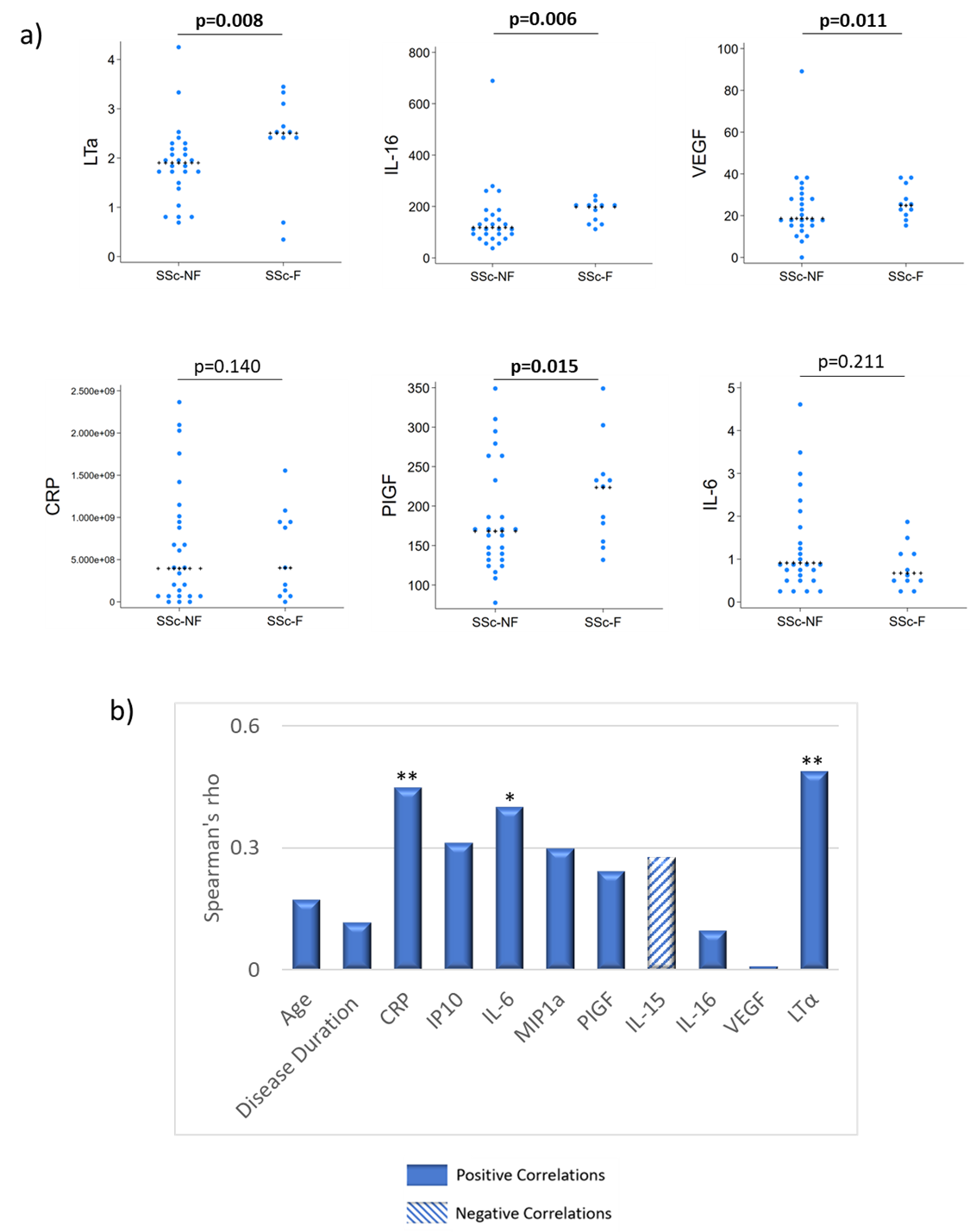


**Supplementary Figure 5: Cytokines and their association with fatigue and Galectin-9**. Cytokines associated with fibrosis and vasculopathy were measured. a) Lymphotoxin alpha (LTα) (p=0.008), Interleukin 16 (IL-16) (p=0.006), vascular endothelial growth factor (VEGF) (p0.011), and placenta growth factor (PIGF) (p=0.015) were found to be elevated in SSc-F patients (n=20), compared to SSc-NF patients (n=17). b) Spearman correlations showing plasma Gal-9 levels in the UofA cohort, correlating positively with inflammatory cytokines; CRP, IL-6, and LTα. Gal-9 levels did not correlate with age or disease duration.

|  | Galectin-9 | Age | Disease Duration | VEGF | PIGF | IL-16 | LTα | CRP | IL-6 | IL-15 | IP10 | MIP1a |
| --- | --- | --- | --- | --- | --- | --- | --- | --- | --- | --- | --- | --- |
| Galectin-9 | 1 |  |  |  |  |  |  |  |  |  |  |  |
|  | . |  |  |  |  |  |  |  |  |  |  |  |
| Age | 0.1742 | 1 |  |  |  |  |  |  |  |  |  |  |
|  | *0.3305* | . |  |  |  |  |  |  |  |  |  |  |
| Disease Duration | 0.1178 | 0.0304 | 1 |  |  |  |  |  |  |  |  |  |
|  | *0.5115* | *0.8656* | . |  |  |  |  |  |  |  |  |  |
| VEGF | 0.01 | **0.0333** | 0.1686 | 1 |  |  |  |  |  |  |  |  |
|  | *0.9556* | *0.8533* | *0.3461* | . |  |  |  |  |  |  |  |  |
| PIGF | 0.244 | 0.2259 | 0.0886 | 0.2433 | 1 |  |  |  |  |  |  |  |
|  | *0.1704* | *0.2051* | *0.6219* | *0.1716* | . |  |  |  |  |  |  |  |
| IL-16 | 0.0976 | 0.2497 | -0.0773 | 0.2259 | 0.5805 | 1 |  |  |  |  |  |  |
|  | *0.5872* | *0.1604* | *0.6672* | *0.205* | ***0.0005*** | *.* |  |  |  |  |  |  |
| LTα | 0.4879 | 0.0873 | 0.1187 | 0 | 0.0924 | -0.0149 | 1 |  |  |  |  |  |
|  | ***0.0044*** | *0.6274* | *0.5084* | *1* | *0.6072* | *0.9342* | *.* |  |  |  |  |  |
| CRP | 0.4495 | 0.0808 | 0.1291 | 0.1855 | 0.36 | 0.3646 | 0.1812 | 1 |  |  |  |  |
|  | ***0.0092*** | *0.6532* | *0.4715* | *0.2997* | ***0.0401*** | ***0.0375*** | *0.3112* | *.* |  |  |  |  |
| IL-6 | 0.4003 | -0.0254 | 0.1594 | 0.2581 | 0.353 | 0.296 | 0.1022 | 0.6793 | 1 |  |  |  |
|  | ***0.0216*** | *0.888* | *0.3734* | *0.1465* | ***0.0444*** | *0.0944* | *0.5695* | *0* | *.* |  |  |  |
| IL-15 | -0.2757 | -0.0661 | 0.1865 | 0.3633 | 0.0084 | 0.2754 | -0.4035 | 0.2594 | 0.2383 | 1 |  |  |
|  | *0.12* | *0.7134* | *0.2968* | ***0.0382*** | *0.963* | *0.1205* | ***0.0205*** | *0.1444* | *0.1807* | . |  |  |
| IP10 | 0.3128 | 0.2269 | 0.1116 | -0.1059 | 0.4328 | 0.4348 | 0.1862 | 0.5084 | 0.3311 | -0.0762 | 1 |  |
|  | *0.0764* | *0.203* | *0.534* | *0.5554* | ***0.0125*** | ***0.012*** | *0.2979* | ***0.0029*** | *0.0601* | *0.6718* | . |  |
| MIP1a | 0.2996 | 0.1981 | -0.0392 | -0.0913 | 0.1491 | 0.2979 | 0.4874 | 0.3889 | 0.3043 | 0.0513 | 0.46 | 1 |
|  | *0.0903* | *0.2677* | *0.8276* | *0.6116* | *0.4055* | *0.0921* | ***0.0044*** | ***0.0259*** | *0.0851* | *0.7755* | ***0.0076*** | . |

**Supplementary Figure 6. Correlates of Galectin-9**. Spearman correlations showing the relationship between Gal-9 and cytokines levels in the UofA cohort.

1. Bedree H, Sunnquist M, Jason LA. The DePaul Symptom Questionnaire-2: A Validation Study. *Fatigue* 2019; **7**(3): 166-79.

2. Smets EM, Garssen B, Bonke B, De Haes JC. The Multidimensional Fatigue Inventory (MFI) psychometric qualities of an instrument to assess fatigue. *J Psychosom Res* 1995; **39**(3): 315-25.

3. Yellen SB, Cella DF, Webster K, Blendowski C, Kaplan E. Measuring fatigue and other anemia-related symptoms with the Functional Assessment of Cancer Therapy (FACT) measurement system. *J Pain Symptom Manage* 1997; **13**(2): 63-74.

4. Ware JE, Jr., Sherbourne CD. The MOS 36-item short-form health survey (SF-36). I. Conceptual framework and item selection. *Med Care* 1992; **30**(6): 473-83.

5. Herrmann C. International experiences with the Hospital Anxiety and Depression Scale--a review of validation data and clinical results. *J Psychosom Res* 1997; **42**(1): 17-41.

6. Buysse DJ, Reynolds CF, 3rd, Monk TH, Berman SR, Kupfer DJ. The Pittsburgh Sleep Quality Index: a new instrument for psychiatric practice and research. *Psychiatry Res* 1989; **28**(2): 193-213.

7. Broadbent DE, Cooper PF, FitzGerald P, Parkes KR. The Cognitive Failures Questionnaire (CFQ) and its correlates. *Br J Clin Psychol* 1982; **21**(1): 1-16.

8. Fries JF, Spitz P, Kraines RG, Holman HR. Measurement of patient outcome in arthritis. *Arthritis Rheum* 1980; **23**(2): 137-45.

9. Bozorgmehr N, Mashhouri S, Perez Rosero E, Xu L, Shahbaz S, Sligl W, et al. Galectin-9, a Player in Cytokine Release Syndrome and a Surrogate Diagnostic Biomarker in SARS-CoV-2 Infection. *mBio* 2021; **12**(3).

10. Saito S, Shahbaz S, Osman M, Redmond D, Bozorgmehr N, Rosychuk R, et al. Diverse immunological dysregulation, chronic inflammation, and impaired erythropoiesis in long COVID patients with chronic fatigue syndrome. *Journal of Autoimmunity* 2024; **147**: 103267.

11. Kim ST, Munoz-Grajales C, Dunn SE, Schneider R, Johnson SR, Touma Z, et al. Interferon and interferon-induced cytokines as markers of impending clinical progression in ANA(+) individuals without a systemic autoimmune rheumatic disease diagnosis. *Arthritis Res Ther* 2023; **25**(1): 21.

12. Chavez-Galan L, Ruiz A, Martinez-Espinosa K, Aguilar-Duran H, Torres M, Falfan-Valencia R, et al. Circulating Levels of PD-L1, TIM-3 and MMP-7 Are Promising Biomarkers to Differentiate COVID-19 Patients That Require Invasive Mechanical Ventilation. *Biomolecules* 2022; **12**(3).

13. Fluge O, Mella O, Bruland O, Risa K, Dyrstad SE, Alme K, et al. Metabolic profiling indicates impaired pyruvate dehydrogenase function in myalgic encephalopathy/chronic fatigue syndrome. *JCI Insight* 2016; **1**(21): e89376.
